# Can computed crystal energy landscapes help understand pharmaceutical solids?

**DOI:** 10.1039/c6cc00721j

**Published:** 2016-04-12

**Authors:** Sarah L. Price, Doris E. Braun, Susan M. Reutzel-Edens

**Affiliations:** a Department of Chemistry, University College London, 20 Gordon Street, London WC1H 0AJ, UK; b Institute of Pharmacy, University of Innsbruck, Innrain 52c, 6020 Innsbruck, Austria; c Small Molecule Design and Development, Eli Lilly and Company, Lilly Corporate Center, Indianapolis, IN 46285, USA

## Abstract

Computational crystal structure prediction (CSP) methods can now be applied to the smaller pharmaceutical molecules currently in drug development. We review the recent uses of computed crystal energy landscapes for pharmaceuticals, concentrating on examples where they have been used in collaboration with industrial-style experimental solid form screening. There is a strong complementarity in aiding experiment to find and characterise practically important solid forms and understanding the nature of the solid form landscape.

## Introduction

Drug molecules are chosen for their biological properties, and their solid form properties have to be exploited or worked around in order to produce the optimum pharmaceutical product. The drug discovery process usually defines the molecule, and the solid form properties of the molecule are later optimised in drug development. The investigation of solid form properties thus has a rather different role in pharmaceutical development than in the design of functional organic materials, where the molecules themselves are ‘optimised’ to achieve key physical properties defined by the crystal structure. Nonetheless, drug development scientists seek to engineer the optimum solid form properties, such as stability, solubility, dissolution rate, and process parameters,^1^–^4^ through considering single and multicomponent crystals, particularly salts and cocrystals, and amorphous forms. The experience of late-appearing, more stable forms, as in the case of ritonavir^5^ or rotigotine,^6^ and the possibility of “disappearing polymorphs”^7^ means that it is essential that the drug product is designed knowing the solubility and other properties of the most stable crystalline form. Many drug molecules have difficulty in crystallising at all, and some ‘metastable’ forms may have better properties, such as solubility, for developing into the drug product. This has led to the development of polymorph screening, a survey of crystallisation conditions designed to identify solid forms of a drug substance and to determine their crystallisation behaviour. Solid form screens may encompass hundreds or even thousands of crystallisation experiments and need to be tailored to the properties of the individual molecule.^8^,^9^ However, drug molecules are typically subjected to solid form screening early in development, when material quantity and/or purity is often limited, and there are pressures on timescales to proceed with biological testing.

Polymorphism, the existence of different crystal structures with the same chemical composition as defined by covalent bonding and stereochemistry, occurs in at least 50% of molecules that have been subjected to industrial polymorph screens.^8^ Multiple solid forms, including salts, cocrystals and solvates, have been found for 90% of molecules,^10^ extending the range of solid form options available for delivering drugs. The second component in salts and cocrystals is generally chosen to improve properties. Moreover, the ubiquity of water in the environment and its presence during processing means that hydrate formation often cannot be avoided and thus the properties of those molecular compounds also need to be established. The (pharmaceutical) solid state may well show further complexity, leading to problems of classification of polymorphism^11^ with there being structural types with continuous variation, such as disorder, solid solutions, even salts/cocrystals with varying proton positions.^12^

Virtually all pharmaceutical molecules contain flexibly linked functional groups with multiple, competing hydrogen bonding and π–π stacking possibilities. The size and flexibility of the molecules currently under development may be more challenging than generic small pharmaceuticals studied in academia. Studies on generic pharmaceuticals such as aspirin, paracetamol and phenobarbital^13^ already show considerable complexity in their polymorphic behaviour, though some polymorphs may be sufficiently short-lived and difficult to observe that they could be considered as being only of “academic” interest. Can we assume that the same principles governing the occurrence of polymorphism and multiple solid forms extend to larger pharmaceutical molecules in current development? One recent survey found no statistical correlation with size or degree of flexibility in the likelihood of a molecule being polymorphic.^8^ Another survey of all small organic molecules (*M*_r_ < 1000) in the European Pharmacopoeia^14^ showed a decrease in the likelihood of polymorphism with increasing molecular weight, though this may simply reflect the difficulty in crystallising larger molecules as solvent free forms. Every polymorph screen is unique^9^ and a matter of observation and experience. The plethora of possible experiments, which today extends beyond conventional methods to include heteronuclear screening using a bank of polymers,^28^ as well as crystallisation in electric^29^ and ultrasound^30^ fields, in laser beams^31^,^32^ and under confinement,^33^ makes it difficult to have a recipe for complete confidence that all possible polymorphs are found. This has led to sustained interest in whether computational modelling can be used to reliably predict polymorphs, their properties and the experiments needed to produce seed crystals.^23^

**Fig. 1:**
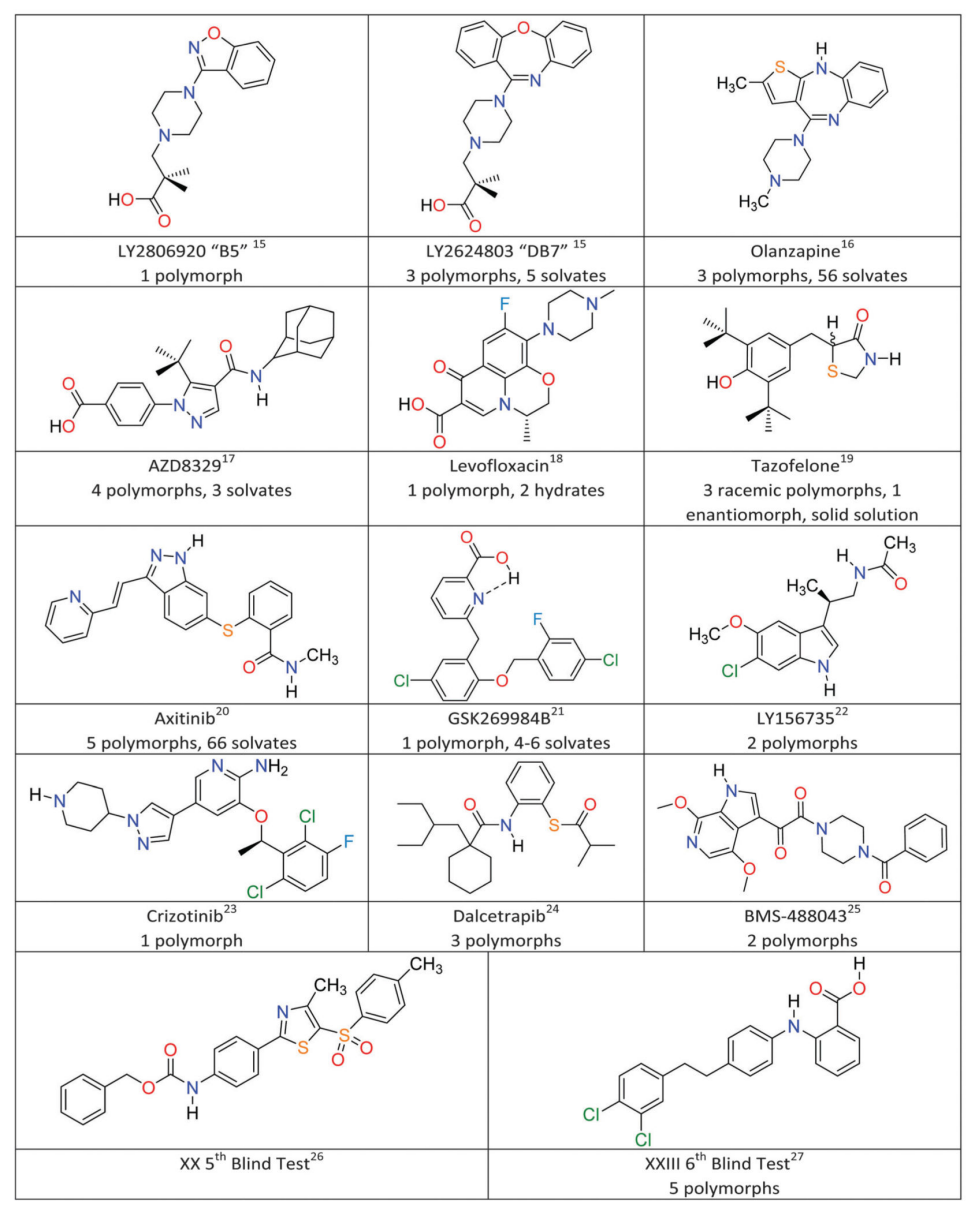
Model pharmaceuticals with combined experimental and computational solid form screening.

The fundamental scientific challenge of whether we could predict crystal structures started in the days when polymorphism was seen as a rarity, and so the approach of searching for the most thermodynamically stable crystal structure became known as crystal structure prediction (CSP).^34^,^35^ As interest in polymorphism increased, it became obvious that some of the low energy structures, which were local minima in the crystal energy, corresponded to polymorphs. To date, crystal structure prediction studies have been performed on many model drug compounds, and in cases such as aspirin, paracetamol, carbamazepine and 5-fluorouracil, have anticipated the discovery of new polymorphs.^36^ The computational challenge of performing CSP studies increases non-linearly with the size and flexibility of the molecule, or the number of independent components in the crystal. Given the potential usefulness of CSP methods to the pharmaceutical and speciality chemical industries, the Cambridge Crystallographic Data Centre has been organising blind tests of whether those developing crystal structure prediction algorithms can predict crystal structures from the chemical diagram since 1999.^37^ The targets have been increasing in molecular size and difficulty reflecting the evolving capabilities of the leading codes, and the 5th Blind Test^26^ included molecule XX (Fig. 1), which was seen as approaching the size of the smaller molecules in drug development. The successful prediction of the crystal structure of XX by two groups^38^ led to various industry/academia collaborations on whether CSP methods would be a useful complement to industrial solid form screening activities. Indeed, the recent 6th Blind Test^27^ included a molecule XXIII (Fig. 1), which had been screened in industry, and the challenge changed to asking participants to submit lists of 100 predicted structures. The account of the 6th Blind Test gives an overview of the progress and range of computational methods being developed to tackle the challenges of covering a sufficient range of crystal structures and of scoring the most likely to be observed, usually done on relative lattice energies.^27^ This feature article covers CSP aided studies on pharmaceutical materials published since the 5th Blind Test, which have been performed in collaboration with industrial, or similar, polymorph screening. Fig. 1 gives the molecular diagrams, along with basic information on the number of polymorphs and other solid forms. What have these CSP studies shown that adds insight to the experimental results?

## Uses of crystal structure prediction in pharmaceutical development

### Finding the most stable form

**Fig. 2:**
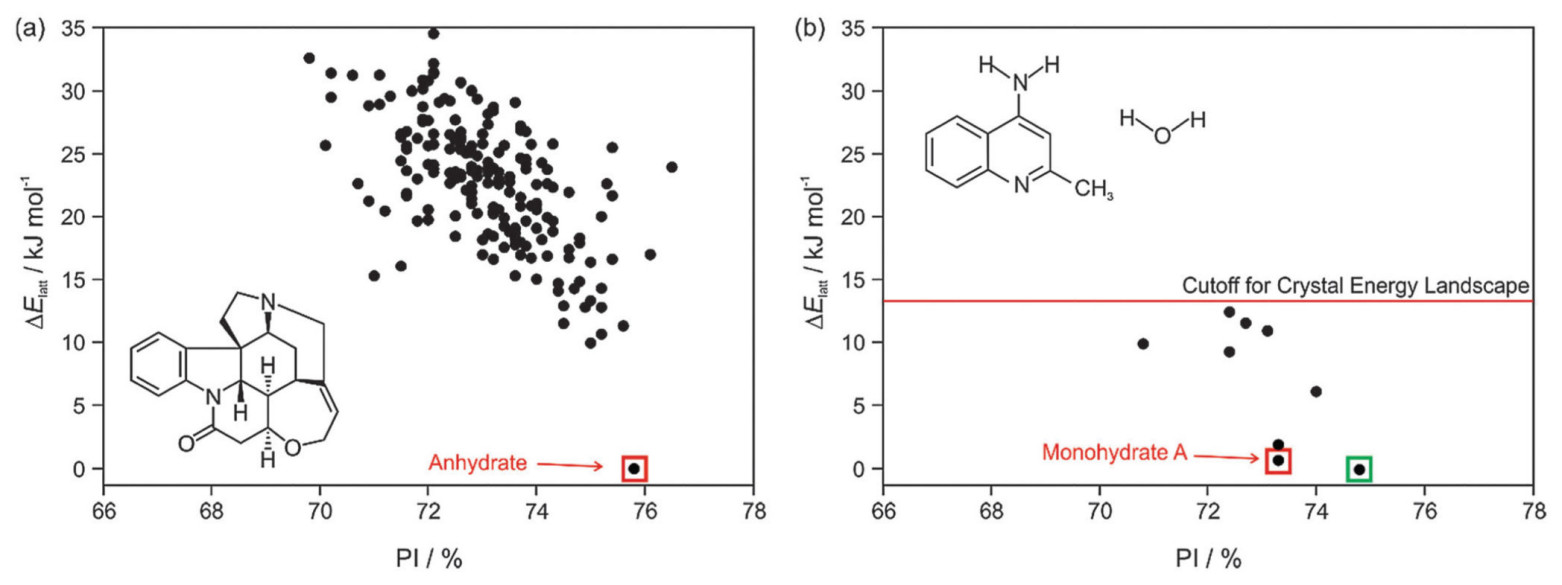
Summary of the CSP study of (a) strychnine (*Z*′ = 1 and 2) and **(b)** 4-aminoquinaldine monohydrate (*Z*′ = 1, adapted from ref. 41). Each point represents the lattice energy relative to the global minimum and the packing index, PI, of a mechanically stable structure, whose full 3D structure file (.res) is generated. The points corresponding to the observed structures are highlighted: red – experimental forms known from prior screening, green – form found guided by the calculations. The structures were generated using CrystalPredictor and all structures shown were refined by periodic electronic structure calculations (PBE-G06), with the energy cutoff for such refinement being shown on (b), to give their relative lattice energies Δ*E*_latt_.

The most important output of a polymorph screen is the most stable form at storage and production conditions, and thus the main hope is that a CSP study would confirm that this is known. In the case of strychnine, the only known unsolvated structure is calculated to be so much more stable than any other, that this confirms the screening result that strychnine is not polymorphic (Fig. 2a). The large energy gap, which need not be calculated to great accuracy, implies that strychnine has a uniquely favourable way of packing defining the crystal structure in all three dimensions into a close packed solid. (The packing index of 76% shows that the molecules are packing more densely than close-packed spheres at 74%). An energy gap of this size would not be affected by the inclusion of temperature effects, and indeed, strychnine does not show any phase changes upon cooling to 15 K^39^ or heating before sublimation, melting or decomposition. Such a large energy gap is relatively unusual, as many molecules can have a preferred conformation and strong interactions, such as hydrogen bonding, defining a strongly preferred ribbon or even layer, but that motif usually can pack in a range of different ways.

A more typical output is shown by 4-aminoquinaldine,^40^ where there were various competitive low energy structures for the monohydrate.^41^ Fig. 2b shows just the crystal energy landscape of those structures which were sufficiently low in energy to be thermodynamically feasible as polymorphs. In this case, the calculations predicted a structure which was slightly more thermodynamically stable and denser than the known structure. Inspired by this prediction, targeted experiments using hydrostatic conditions (crystallisation at higher temperatures under elevated pressure in a hermetically sealed DSC pan) led to the most stable form of 4-aminoquinaldine monohydrate. This form had proven very difficult to access experimentally for kinetic reasons, though it could also be crystallised from selected solvents at normal pressures if a chemically-related phase impurity, chloro-4-aminoquinaldine, was present.^41^

Another case where the calculated crystal energy landscape predicted that a more stable polymorph existed was creatine,^42^ a zwitterionic food supplement. The long-known form was a metastable form but with extremely high kinetic stability (and an earlier CSP study with too limited a search had concluded^43^ that this would be the only form). A more recent CSP study found two thermodynamically competitive structures,^42^ which were both found in two independent experimental screens.^42^,^44^ Creatine and 4-aminoquinaldine monohydrate represent cases where careful experimentation has been able to find the most stable form guided by observation and the CSP generated structures, and all the energetically most competitive CSP generated structures have been observed. Whenever there are very thermodynamically competitive structures, the question naturally arises as to whether these polymorphs could be found.^45^

### Finding all relevant polymorphs

**Fig. 3:**
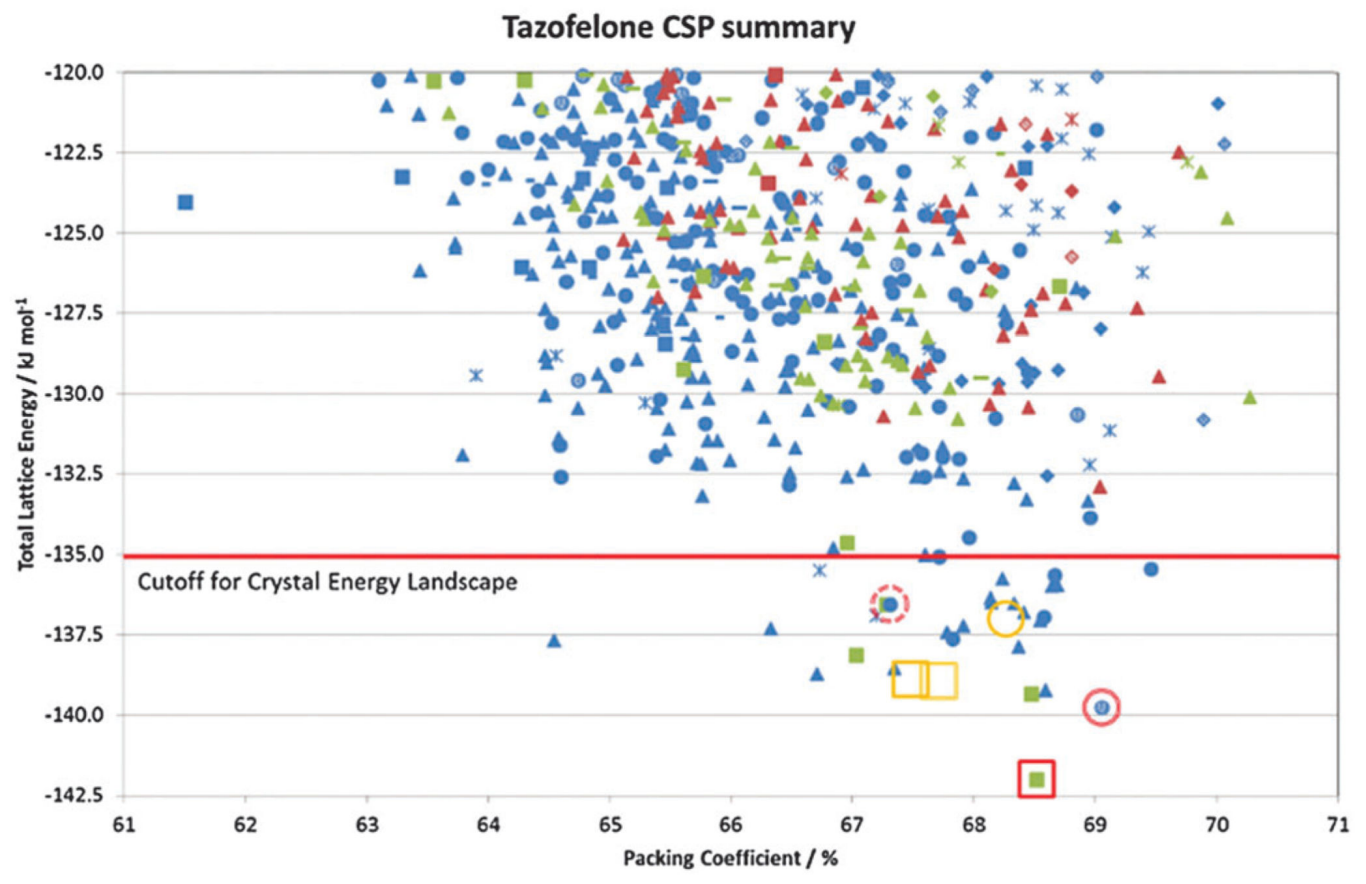
Summary of the CSP study of tazofelone adapted from ref. 19. Each point represents a lattice energy minimum, with structures categorised by colour to denote the type of conformation (AB extended, blue; F folded, green; C another low energy conformation, red) and symbol shape to denote the hydrogen bonding graph set, some of which are defined in Fig. 4. Open symbols denote the corresponding lattice energy minima for the experimental structures (labelled on Fig. 4) which are red for *Z*′ = 1 structures included in the search and orange for *Z*′ = 2 structures which are beyond the scope of the search.

In many cases, the most stable form is found as the most stable structure, at least within the uncertainty in the calculation of the relative energies, and then the question becomes which of the structures on the crystal energy landscape would seem likely to be practically important polymorphs, and how might they be found. This requires looking at the structures to see similarities between them using various tools such as hydrogen bonding graph sets^46^ and other structure comparison tools.^47^,^48^ A summary of the output of a CSP study for tazofelone^19^ is shown in Fig. 3, which shows that the “C” conformer cannot pack to give thermodynamically plausible structures, and that the other types of conformers can only pack with a few types of hydrogen bonding motif to give structures of sufficient thermodynamic stability to be considered as potential polymorphs. It is this type of classification and comparison with the packing of the drug molecule and closely related molecules in all their known forms (as an expansion of, for example, the analysis of 50 carbamazepine-containing crystal structures^49^) that can provide the most insight into the crystallisation behaviour required for drug development.

### Suggesting experiments to find new polymorphs

A polymorph that had not been identified in extensive experimental screening of Roche’s CETP inhibitor, dalcetrapib, has recently been found by crystallisation under pressure, an experiment suggested by the CSP study.^24^ The crystal energy landscape showed two structures very close in energy, but denser than the known stable form, and the unknown structures were calculated to become more stable than the observed polymorph with a modest increase in pressure. Experiments recrystallising dalcetrapib, either from solution or the melt in a diamond anvil cell under modest pressure formed a new polymorph. This matched the predicted polymorph, except for disorder in the hydrocarbon tail, which could have been anticipated from the related structures on the crystal energy landscape.

The known polymorphs of the extensively-screened pharmaceutical carbamazepine were, until recently, all based on hydrogen bonded dimers, although CSP studies suggested that structures based on a hydrogen bonded catemer were at least thermodynamically competitive. As carbamazepine was often used as a test case for solid form screening, and indeed the discovery of Form IV was an early success of polymer heteroscreening,^50^ the kinetic accessibility of a catemeric polymorph was debated.^51^ Eventually the catemeric carbamazepine polymorph Form V was found by subliming the drug onto a crystal of dihydrocarbamazepine Form II, which was isostructural with the targeted polymorph.^52^ This principle of templating by isostructural solid forms has also been demonstrated for a predicted polymorph of cyheptamide.^53^ A CSP predicted benzoic acid:caffeine cocrystal similarly proved to be elusive^54^ until seeded with a structurally related molecule, an experiment which was repeated in four geographically distinct laboratories. These examples show how having CSP predicted polymorphs can suggest heterogeneous seeding experiments for cross nucleating polymorph(s), though far more understanding of heterogeneous nucleation is required before such tailored experiments can be routinely applied in pharmaceutical development. Nonetheless, it is well recognised that impurities can play a major role in determining which polymorphs can be crystallised^7^ and hence spiking with possible reaction product impurities is often included in exhaustive polymorph screening.

### Determination of crystal structure when single crystals cannot be grown

An important use of the CSP study during a polymorph screen is to help determine and confirm crystal structures from experimental powder diffraction data, when there are no single crystals suitable for X-ray diffraction. The CSP generated structures can suggest possible trial structures to be refined against the experimental data or simply add confidence or detail to the proposed structure. The calculation of the powder diffraction spectrum from a CSP generated crystal structure with atomic coordinates is trivial, requiring as input only the wavelength of radiation. However, the powder pattern is very sensitive to the cell parameters and the anisotropic thermal expansion of organic crystals, and CSP generated structures will be in error typically by a few %, comparable to the neglected thermal expansion. Methods are being developed to facilitate the automatic use of CSP structures to help solve structures from powder data.^55^ There are many reported CSP-aided structure solutions from powders, ranging from cases where the powder pattern could not be indexed,^56^ through suggesting a set of possible structures that are consistent with pattern indexing, as discussed later for metastable forms of olanzapine^16^ and DB7,^15^ to correcting the proton positions.^57^ Transmission electron microscopy and electron diffraction have also been used to detect and characterise new polymorphs and propose their structures by comparison with CSP generated structures,^58^ allowing structural characterisation of a polymorphic impurity that was undetectable by powder X-ray diffraction.^59^

The ability to calculate solid state NMR spectra from CSP generated structures is increasingly being used for “NMR crystallography”.^60^,^61^ One application to pharmaceutical development is AZD8329, an 11β-HSD1 inhibitor investigated for use in the treatment of type 2 diabetes. A structure for AZD8329 Form 4, one of two forms considered to have superior properties for development, was proposed^17^ by comparing the experimental proton solid state NMR spectrum with those calculated from CSP generated structures. This structure for Form 4 was in excellent agreement with that independently determined from powder X-ray diffraction, with the advantage of NMR spectroscopy having located the carboxylic acid proton position.

When CSP is being used as an aid to structure determination, it is possible to restrict the search space using experimental data. For example, a plausible model for the structure of the antibiotic levofloxacin was proposed after using the crystal structures of six carboxylic acid salt and hydrate forms to identify six likely π⋯π stacked dimer structures, which were optimised by electronic structure methods and held rigid during the CSP search.^18^

### The nature of the solid form landscape

The outcome of a CSP study is a set of 3D crystal structures of the molecule(s) and an estimate of their relative stability. The interpretation of the results can be straightforward, as in the crystal energy landscape of strychnine (Fig. 2a), or there may be a variety of different aspects to the crystallisation behaviour revealed from careful analysis of the structures, as shown for tazofelone.^19^ The following examples show how the different crystal energy landscapes can help rationalise the differences in crystallisation behaviours that are often an issue in (drug) development.

### Solid form diversity, disorder and why mixtures of polymorphs sometimes cannot be avoided

In the CSP study of 5-HT_2a_ agonist DB7 (Fig. 1),^15^ a metastable polymorph, Form III, could only be obtained in polycrystalline form by desolvating the zwitterionic dihydrate.^62^ The question was raised as to whether sample-to-sample variability in the properties of Form III was due to concomitant dehydration to two closely related polymorphs. The computed crystal energy landscape found a match to its powder diffraction pattern in two structures, differing only in the propionic acid conformation, allowing characterisation of this solid form as a single polymorph with variable sidechain disorder. In this instance, the combined use of experimental screening and CSP showed a disordered structure for Form III was inevitable, helping to clarify the number of forms produced by the solid form screening.^15^

The neat form of orotic acid is another case where the anhydrate has different levels of order–disorder, this time in the stacking of a layer structure, depending on how the very stable hydrate or non-layer dimethylsulfoxide monosolvate are desolvated. Packing comparisons and chemical shift calculations for layer structures on the computed crystal energy landscape provided models for the stacking faults.^63^

**Fig. 4:**
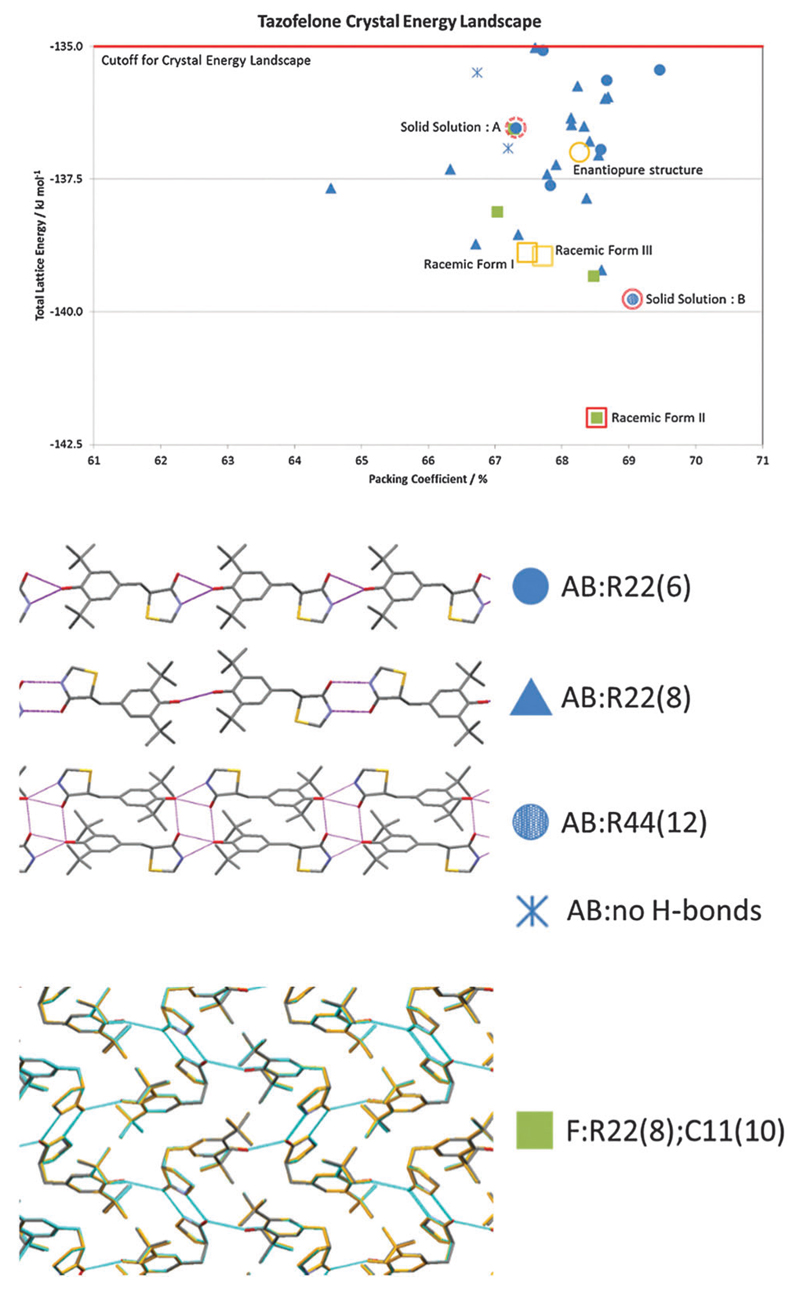
The crystal energy landscape for racemic tazofelone, with the key to the hydrogen bonding motifs. The figure for F:R22(8);C11(10) is an overlay of this sheet in the three racemic polymorphs, adapted from ref. 19.

The problems of differentiation between polymorphs and degrees of disorder, and the consequences for the quality control of crystal properties, is further exemplified by the case of tazofelone. The original screening of racemic tazofelone had produced two polymorphs,^64^ which were based on the same F:R22(8);C11(10) layer structure (Fig. 4) with different stackings in the third dimension. Revisiting this compound to obtain good thermodynamic data for calibrating the CSP study^19^ unexpectedly produced an alternative stacking as a third polymorph. A particular concern for ensuring quality control over material properties was that the large single crystals of each polymorph varied in melting point! A detailed examination of the *hkl* raw diffraction images showed some streaking, characteristic of significant stacking disorder within the crystals which accounts for the crystal specific properties. The crystal energy landscape (Fig. 4) had the most stable form as the global minimum, but showed that there were other ways of stacking the layers that were so close in energy, that stacking errors or different polymorphic domains (polytypes) within single crystals were probably unavoidable.

The problem of disorder *versus* concomitant crystallisation is also apparent for olanzapine, where claims were made for novel forms that were mixtures of the concomitantly crystallising metastable polymorphs, Forms II and III. The structure of Form was only determined in 2011, when a single crystal suitable for X-ray diffraction analysis could be picked out from a sample of olanzapine that had failed to co-crystallise with nicotinamide.^65^ A single crystal suitable for X-ray diffraction experiments of Form has not yet been found; however, the crystal energy landscape included a structure that was a sufficient match to the Form III powder pattern to show that it was a different stacking of the same molecular layers as Form II.^16^ This structural model rationalises why Forms II and III crystallise concomitantly, with it being practically impossible to generate phase pure samples.

In these cases, the structures on the crystal energy landscape show how little energy discrimination there is between closely related structures, thus revealing the possibility of disorder (conformational or stacking faults) rather than varying proportions of different phases. These systems also illustrate the continuum between closely related polymorphs and varying degrees of disorder.^12^ Static disorder can be a thermodynamic effect: generating all the ordered structures for the 20 molecule unit cell of the low temperature form of caffeine (Form II) showed that static disorder was favoured by configurational entropy.^66^ However, only some of the contributing structures appeared in the crystal energy landscape for caffeine generated by assuming that all the molecules were related by the space group symmetry operations. Hence, scientists have to use their experience to interpret the experiments and crystal energy landscape to estimate whether closely related calculated structures are likely to be seen as disorder, or alternatively, are so similar that they would readily transform to the most stable structure during the crystallisation process, or not be separate free energy minima at normal temperatures.

### Why are some molecules prolific solvate formers?

A CSP search only generates idealised crystal structures of the input molecules, and yet can help show the reasons behind solvate formation. It can show that a molecule cannot pack densely by itself, and hence there will be a tendency for solvent to fill the voids in the structure and stabilise it through non-specific dispersion interactions. This can lead to isostructural solvates, where different solvents or mixtures can be in crystal structures which are virtually identical in the packing of the drug molecule. CSP can generate the guest-free framework of inclusion compounds as a low density structure.^67^ More specific solvate formation often occurs for pharmaceuticals when the hydrogen bonding sites are satisfied by water/solvent molecules, particularly for drugs where the number and disposition of the hydrogen bond donors or acceptors means that they cannot all be involved in hydrogen bonding.

Many pharmaceuticals are prolific solvate formers, with sulfathiazole having over 100 solvates reported.^68^ This considerably complicates the solid form screening output. Pharmaceutical solvates with solvents which are not suitable for pharmaceutical processing, as they are not on the GRAS (Generally Recognized as Safe)^69^ list, have to be considered in screening because desolvation^20^ is a sufficiently productive method of finding new forms and may be the only route to a new polymorph.^40^,^42^,^62^,^70^ Once the first sample is obtained, further samples can be produced by seeding, either intentionally or unintentionally. Unlike organic solvates, all possible hydrates have to be identified and their (de)sorption behaviours extensively studied and characterised because of the difficulty of rigorously excluding water from production processes. Solvates can include multiple solvents, sometimes in variable ratios, and the distinction between surface bound water, stoichiometric and non-stoichiometric hydrates is critical for process design and can be difficult to establish.^62^ Labile solvates, where the solvent readily leaves the crystal when it is removed from the crystallising solution, are common. This can lead to highly metastable forms if there is a large kinetic barrier to rearrangement. If no solvates are formed then this may reflect the ease with which the non-solvated form crystallises, unless the range of screening experiments is limited, for example, by the solubility of the crystalline form.

Olanzapine illustrates how the inability of a molecule to pack well with itself can give rise to a multitude of solid forms, with over 60 being found in the screen.^16^ Many of them were isostructural solvates having differing solvent mixtures between layers of olanzapine dimers, and the crystal energy landscape showed that these layers do not stack particularly well to form an unsolvated crystal. The separation of solvate motifs and polymorphs is more challenging for the Pfizer oncolytic axitinib (AG013736), which has 71 solid forms, including 5 polymorphs containing just the drug molecule.^20^ A CSP study^71^ found all of the axitinib polymorphs, but also showed that there are many alternative structures that were thermodynamically competitive. Considerable efforts had already gone into developing targeted screens^20^ to circumvent the solvation issues associated with conventional screening methods. Hence the expense of further work would only be justified if a clear pathway to crystallising further non-solvated polymorphs of axitinib could be proposed.

### Why do molecules not crystallise at all/form a stable amorphous phase?

A major complication in pharmaceutical development is when a molecule fails to crystallise readily, or at all, instead forming an amorphous phase. The amorphous form, with its greater solubility, could be an attractive alternative for delivering a poorly soluble drug if it could be relied upon to not crystallise. However, experimentally concluding that an amorphous form is stable is difficult, given the problem of late appearing polymorphs. There have been informatics methods developed to seek a statistical probability of a compound not crystallising^72^,^73^ that are based on molecular descriptors and assumptions in classifying the training dataset with respect to the effort that has gone into trying to crystallise a molecule. The motifs generated in a CSP study have provided some rationalisation, for example, why one molecule forms a gel and its isomer crystallises. In this case, the CSP study predicted the crystalline solid structure and suggested that the packing preference for the gel former was one-dimensional hydrogen bonding arranged into tightly coiled molecular columns which could pack in many ways.^74^ A CSP study of salicylsalicylic acid (salsalate), a molecule widely studied for the stability of its amorphous phase, generated a variety of energetically competitive structures, based on different hydrogen bonding chains and other motifs.^75^ The different hydrogen-bonded chains identified in the CSP-computed structures appear to be seriously detrimental to the molecule’s ability to pack efficiently and stably with the internal hydrogen bonding that is seen in the experimental crystal structure. The CSP structures provided a good basis for a model of the amorphous phase; however, the experimental analysis showed that amorphous salsalate is prone to contain oligomeric thermal decomposition products that could also frustrate crystallisation, and it was possible to crystallise salsalate more readily than had previously been suggested.^75^

### Why do structurally similar molecules from the same drug discovery program have very different crystallisation behaviour?

It might be expected from drug design principles that molecules which are similar enough to bind to the same receptor would also have similar modes of self-recognition and hence crystal structures. However, molecules from the same drug discovery program can pose very different challenges when it comes to progressing solid forms in drug development. A CSP study investigating this used two 5-HT_2a_ agonists, LY2806920 (B5) and LY2624803 (DB7) (Fig. 1), which contain virtually the same hydrogen bonding groups and were both under development for sleep disorders. B5 readily and reliably crystallises into just one solid form containing the neutral molecule, whereas three neat polymorphs, two hydrates, three alcohol solvates and an amorphous phase are known for DB7.^15^ The difference in the crystallisation behaviour was not a function of the distribution of the energies of alternative structures. B5 was able to pack densely with itself, and most of the energetically competitive structures have the same internal hydrogen bond as the readily crystallising structure. In contrast, DB7 was only able to crystallise in structures which had relative low packing efficiencies, and the structures that were competitive with the most stable form had a variety of intermolecular hydrogen bonding motifs. The other known polymorphs were calculated to be relatively unstable, but this is consistent with these polymorphs being produced by dehydration and salt disproportionation. This pair of molecules also illustrates the problem of performing a comparable screening effort: since B5 did not produce any solvates, desolvation studies were not possible, and its ready crystallisation meant that there was no amorphous form to be used as input material.

The difference in crystallisation behaviour between closely related molecules/isomers is common: structural systematics comparing structures with minor changes in functional groups that do not affect the dominant hydrogen bonding motif, can show significant variations in crystal structure, for example in 5-substituted uracils,^76^ mandelic acids,^77^ and 4,4′-disubstituted benzenesulfonamidobenzenes.^78^ Isomers frequently differ: caffeine has two forms, one statically, the other dynamically disordered, whereas isocaffeine is monomorphic,^66^ and 2,4-dihydroxybenzoic acid forms hydrates and its 2,5- isomer does not^79^ as a consequence of the stability of the anhydrous form. Crystal structures are clearly very specific to the individual molecule.

### Aiding the design of chiral separation by crystallisation?

Crystallisation is often seen as an ideal process for the separation of enantiomers, which is required to satisfy regulatory demands that chiral drugs are administered in an optically pure form.^80^ As such, screening and development are generally directed to just the active enantiomer. The dangers of this strategy are shown by LY156735, a melatonin agonist, in which two polymorphs are known, but the most stable form has only been obtained for the inactive *S* enantiomer. In fact, had crystallisation studies not been performed on the inactive isomer, the screening would give no indication that a more stable crystal form existed.^22^ CSP would, however, have alerted the scientists to this possibility, with the crystal energy landscape having both enantiomorphs and the known racemic form within the top 9 structures, all within 1 kcal mol^−1^ of the unobserved most stable form.^22^

**Fig. 5:**
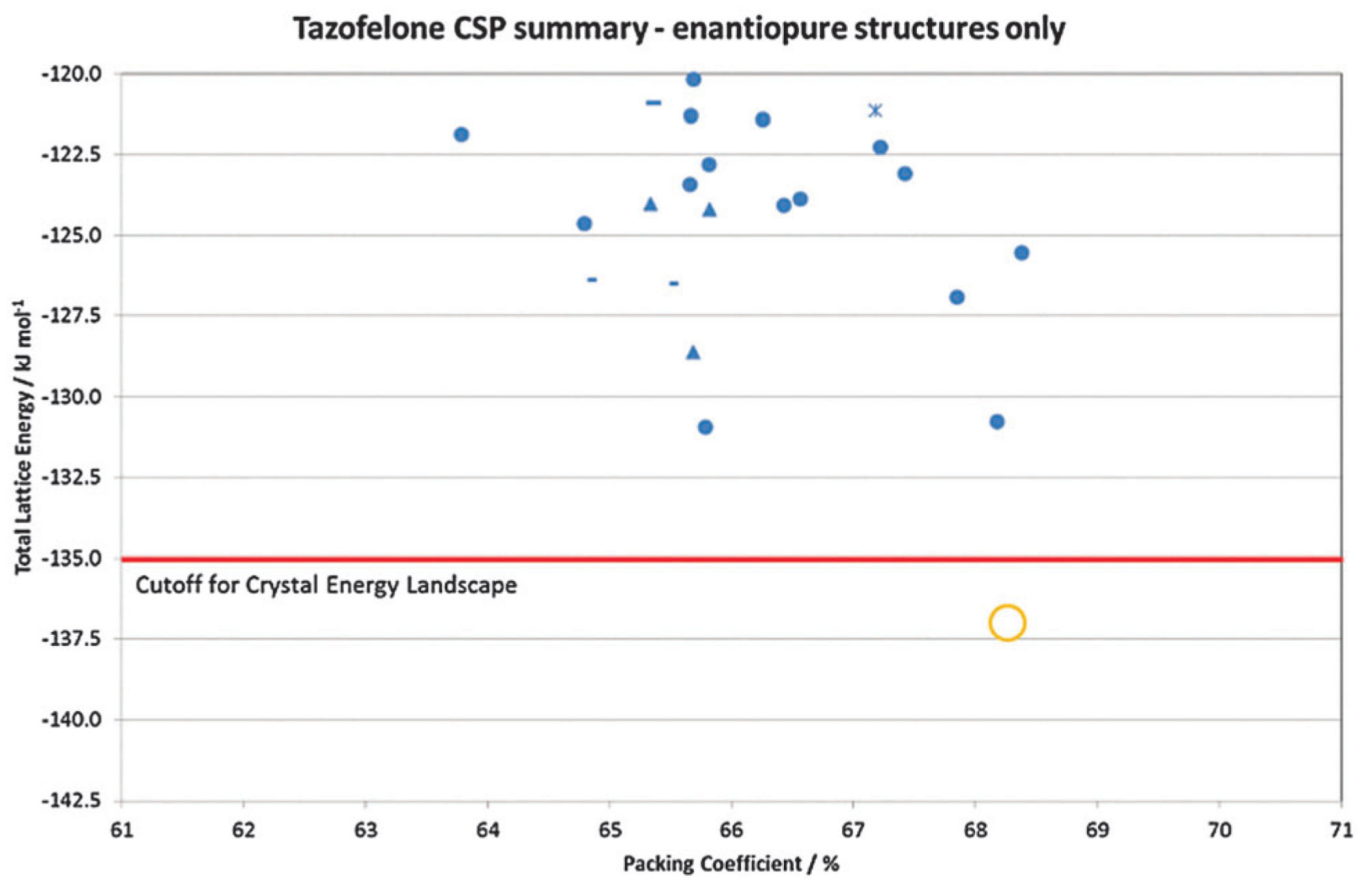
Summary of the CSP generated enantiopure structures for tazofelone adapted from ref. 19. The experimental structure is *Z*′ = 2 and hence not found in the *Z*′ = 1 search (*Z*′ is the number of independent molecular conformations in the crystal.) The horizontal line is the cutoff used for the crystal energy landscape (Fig. 4) that was applied to the CSP summary (Fig. 3) for crystal structures that could be obtained from a racemic mixture.

Chiral resolution by crystallisation is, of course, only possible when the opposite enantiomer is rejected during crystal growth. For tazofelone, the CSP results for enantiopure crystals (Fig. 5) showed that it cannot pack using the expected amide–amide N–H⋯O hydrogen bonding, thus relieving the need for the additional screening suggested^19^ by the CCDC solid form informatics hydrogen bond propensity tool.^81^ Since the observed structure had two conformations of the molecule in the same hydrogen bonding motif (AB:R22(6) Fig. 4) and yet was significantly more stable than the computer-generated structures containing just one conformation, there are not expected to be practically important polymorphs of enantiopure tazofelone. However, the crystallisation of enantiopure tazofelone is not straightforward. The unusual experiment of seeding a racemic melt with the enantiopure (*R* or *S*) crystal instead results in an isostructural solid solution,^82^ *i.e.* the enantiopure crystal structure can include a variable proportion of molecules of the other hand. This phenomenon can be explained by the computed crystal energy landscape^19^ (Fig. 4) including an isostructural racemic structure that is more stable than the enantiomorph, albeit metastable relative to the other known racemic polymorphs. Since a change in conformation of the molecule has the same effect on packing as a change in chirality, recrystallisation of predominantly enantiopure tazofelone will absorb rather than exclude chiral impurities.

## Is the lack of observed polymorphs in a screen reliable?

The failure of experimental solid form screens to produce more than one crystal form may be due to one form being much more stable or crystallising much more rapidly than all others. For true polymorphs, CSP can uniquely suggest whether monomorphism is a product of thermodynamics or crystallisation kinetics. An example where alternative crystal structures were calculated^21^ to be only slightly less stable than the only readily crystallised form is GSK269984B (Fig. 1). In this case, the hypothetical polymorphs had intermolecular hydrogen bonding compensating for adopting grossly different, higher energy conformations than the observed more stable, internally hydrogen bonded conformation.^21^ Further screening, concentrating on solvents that would be likely to hydrogen bond to the drug molecule, produced some metastable solvates with the expected intermolecular hydrogen bonding, but the same gross conformation as in the neat form. Thus the question arises as to whether the fast crystallisation of GSK269984B into its most stable form could be relied upon to prevent the crystallisation of the alternative computer generated structures,^21^ given that solution NMR showed that a range of other conformations could exist in solution. In ritonavir, it was the small solution population of the higher energy conformation that was found in the most stable polymorph that rationalised its disastrous late appearance.^83^,^84^ The key difference for GSK269984B is that the higher energy conformers are calculated to give metastable polymorphs.

Crizotinib was developed by Pfizer for the treatment of forms of lung cancer, and extensive polymorph and hydrate screening similarly found only one crystalline form. A simple CSP search, based on just four rigid, carefully selected conformers and the five most common chiral space groups, showed that the known structure was significantly more stable than any other generated, rationalising the lack of polymorphs.^23^ That the known structure not only had the lowest energy conformation but also optimal intermolecular interactions was confirmed by a CCDC solid form informatics “healthcheck”.^85^ It is unusual that there are no signs of alternative crystal forms in the screening and so the computational confirmation that there is no compromise between conformation and intermolecular packing in the structure, and that it has a uniquely favourable packing defining all three dimensions, provides valuable reassurance.

## Is the number of possible polymorphs unlimited?

Some intensively studied, highly polymorphic molecules, such as the precursor of olanzapine known as ROY^86^ for the red-orange-yellow spectrum of its many polymorphs, or axitinib, have a crystal energy landscape^71^,^87^ where there are a large number of thermodynamically competitive but unobserved polymorphs. Other families, such as the fenamates and barbiturates, are also prone to polymorphism, with flufenamic acid until recently holding the record for the number of solved crystal structures of different polymorphs.^88^ The crystal energy landscapes of the fenamic acids are sensitive to the substitution pattern.^89^ In the case of phenobarbital, with 11 known polymorphs (though five can only be obtained by isomorphic seeding with other barbiturates^90^), four solvates^91^ and two hydrates, CSP suggested^13^ that many further polymorphs are possible, with Form X being later identified as one of the predicted structures.^92^ There are many reasons why CSP often generates more thermodynamically plausible structures than known polymorphs,^45^ but in these cases more polymorphs may be found with better techniques for trapping short lived, metastable forms, or more sophisticated analytical techniques for detecting phase impurities. This means that new forms are still being found for heavily studied ‘old’ molecules. In the cases where CSP studies generate a large number of structures on the crystal energy landscape, McCrone’s famous statement^93^ about the number of polymorphs being determined by the effort expended on looking for them, continues to hold.

## Discussion

### What are the advantages of using CSP in developing pharmaceutical materials?

A CSP study shows what types of crystal packing are particularly favourable for a specific molecule. Crystal engineering principles or informatics-based healthchecks^85^ can quickly show what may be expected for the functional groups within the molecule. However, the vastly more computationally expensive CSP shows the compromises between close packing, conformational preferences and the different types of intermolecular interactions that determine the crystal structures possible for a specific molecule. Thus CSP may generate unexpected and correct crystal structures, as shown in the case of 1-benzyl-1-*H*-tetrazole, testing an unusual functional group sometimes used in pharmaceutical design. The observed crystal structure was unusual and totally different from the tetrazole layer expected from an analysis of similar crystal structures in the Cambridge Structural Database.^94^ Alternatively, a CSP study can show that a molecule cannot pack with the most favourable hydrogen bonding motif in a dense fashion with translational symmetry (*e.g.* enantiopure tazofelone in Fig. 5).^19^

The question of “Are crystal structures predictable?”^27^,^45^,^95^–^97^ periodically comes up as CSP methods continue to improve and this has serious implications for the intellectual property value of crystal forms. The calculations are closing in on predicting thermodynamically feasible packings for a growing number of pharmaceutical compositions; however, the accuracy of the energy calculations, particularly at relevant processing and storage temperatures, and the inability to target any low energy structure on a crystal energy landscape in crystallisation, means that crystal structure prediction in the truest sense (from molecular structure to material in hand) is not yet possible. However, as the examples illustrate, once a CSP study has determined the set of thermodynamically plausible structures for a molecule, then their interpretation in conjunction with the experimental screening data may generate hypotheses for the factors affecting its crystallisation and potential further polymorphs. This can be tested by directed experimentation, if deemed worthwhile, or used to estimate the risks and uncertainties involved in the material properties. There is a long way to go before key properties, such as the solubility, morphology and mechanical properties of different polymorphs at process-relevant temperatures can be estimated reliably from both observed and computer generated crystal structures,^3^ but this could lead to targeted finding of a particular polymorph for its properties.

### What are the challenges in developing CSP as a complement to solid form screening?

The calculation of crystal energy landscapes to a worthwhile accuracy is far from routine even for small molecules, as illustrated by the recent 6th Blind Test of CSP.^27^ Algorithmic developments are on-going particularly to deal with flexibility in the generation of putative crystal structures, but this requires validation data^98^ which the pharmaceutical industry is uniquely suited to provide, as shown by the value of BMS-488043^25^ for the development of CrystalPredictor.^99^ One difficulty of CSP is the vast number of possible structures that need to be considered, as the study will only generate crystal structures with the input molecular connectivity and stoichiometry, and the user-specified range of space groups and number of independent molecules in the unit cell (*Z*′). This latter variable is important: it is far more expensive to cover the search space when there are the additional variables defining the relative position of two or more independent molecules in the asymmetric unit as for cocrystals, salts or solvates. For a single component search, a *Z*′ = 2 search should duplicate the structures found in a *Z*′ = 1 search, but may generate others, which may be closely related to a *Z*′ = 1 structure, or could be intrinsically different, for example when the two molecules are involved in different hydrogen bond interactions, or have different conformations (as in enantiopure tazofelone, Fig. 5). Unfortunately the incidence of *Z*′ > 1 structures for known polymorphic systems is about 20%, almost double that for all crystal structures^8^ within the Cambridge Structural Database. A further choice is often how much molecular flexibility to consider in the search to ensure that all conformational polymorphs could be generated.^100^ The number of possible local minima in the conformational energy rises very sharply with the number of flexible torsion angles. This is a major reason why CSP for pharmaceuticals is so much more demanding than for other types of molecules with less flexibility. Molecules comprised of aromatic groups flexibly linked have a tendency to crystallise in an extended conformation,^101^ as this often allows a denser packing stabilised by the dispersion forces, whereas the more stable isolated molecule conformations with stronger intramolecular interactions often have awkward shapes that cannot pack densely. These and many other possible compromises between the inter- and intra-molecular contributions to the lattice energy have to be explored in the CSP search. Once packed into a crystal, further structural optimisation can only refine the conformation, not cross large energy barriers. However experimentally there is a similar difficulty in transforming between conformational polymorphs, resulting in greater energy differences between conformational polymorphs than for those where the conformations have a common nearest conformational energy minimum.^8^ Hence, an extensive CSP study shows which conformations can generate stable crystal structures. This contributes to the investigation of the extent to which the conformational behaviour in solution, and the mechanisms by which solvent is expelled during nucleation and growth, determine conformational polymorphism.

All CSP methods that have been successfully applied to pharmaceuticals use a relatively cheap method of evaluating the lattice energy in the initial search,^99^,^102^ eliminate duplicate structures and then use more accurate evaluations of the lattice energy. The most accurate methods are used to determine the crystal energy landscape, the set of structures that are sufficiently thermodynamically stable that they may be experimentally accessible. The most successful methods make extensive use of electronic structure calculations, either on the molecule or on the crystal structures. Quantum mechanical calculations on the molecule can estimate the conformational energy of the molecule and provide a conformation-dependent, atomic multipolar model of the charge density for evaluating the electrostatic component of the intermolecular lattice energy.^103^ The other contributions to the intermolecular lattice energy may be evaluated from an empirically fitted transferable model, usually an atom–atom *exp-6* repulsion dispersion model, or a specifically derived model intermolecular potential. Periodic electronic structure methods are usually based on density functional theory (typically the PBE functional) with an essential correction to model the dispersion interaction, either one specifically designed for molecular crystals,^104^ or one of the many being developed.^105^,^106^ The 6th Blind Test showed how predicting relative energies of crystal structures is really challenging the development of computational chemistry methods.^27^ The successful methods used hundreds of thousands of CPU hours. The scaling of the cost of the *ab initio* methods with size of molecule and the scaling of the search space with the number of rotatable torsion angles, means that current methods could not be scaled to a molecule like ritonavir.^5^

A fundamental limitation of the current methods is that they only calculate lattice energies. The ideal crystal energy landscape for thermodynamically plausible structures would be a landscape of the free energy at ambient temperature and pressure. Many lattice energy minima are not free energy minima and the degree to which the dynamic motions average over multiple minima depends on the barriers between the structures. Although free energy can be estimated based on the harmonic approximation, this does not show when a molecule may undergo a transition to a dynamically disordered structure, or one where some functional groups are undergoing large amplitude motions, such as freely rotating methyl groups. The accurate determination of the crystal free energy landscape at ambient temperature and pressure for even the smaller pharmaceuticals represents a significant challenge to computational modelling.^107^ There is a great need for molecular dynamics simulations for both improved thermodynamics and to start to model the kinetics involved in crystallisation. CSP is in active development for greater accuracy, realism and reduced resource requirements, as shown by the range of approaches used in the recent Blind Test of crystal structure prediction.^27^

### Will polymorph prediction ever be a black-box computational tool? Fundamental understanding of crystallisation for flexible molecules

Even if we could calculate an accurate free energy landscape of the possible crystal structures, there is the question of whether we should expect to be able to find all the really distinct but thermodynamically indistinguishable structures on a crystal energy landscape as polymorphs, *cf.* examples of 4-aminoquinaldine (anhydrate and monohydrate)^40^,^41^ and creatine.^42^ Indeed, it is questionable whether we should always be able to crystallise the most stable form, if nucleating the structure is statistically unlikely^77^,^108^ or rearranging the molecules into this structure from solution or solvated prenucleation clusters is unlikely. The case of ritonavir,^5^ where the late appearance of the most stable form has been linked to only 1% of the molecules in solution being in the required conformation, or seeding by an impurity, illustrates the need for both CSP and a better understanding of the competition in the kinetics of nucleation and growth between polymorphs.

As with smaller molecules, the challenges in interpreting the computed crystal energy landscape are to predict which structures are going to be practically important polymorphs, suggest experiments to find them, and design suitable processing methods.^109^ In addition, for larger molecules, with increasing flexibility,^110^ there can be the possibility of trapping highly metastable polymorphs, or difficulty in crystallising the molecule at all. Larger molecules are more liable to thermal decomposition, and may not have sufficient solubility in a wide range of solvents, thus reducing the scope of conventional screening methods. It can be difficult to ensure that a solution is free of nuclei of the input material.^111^ On the other hand, a better understanding of heterogeneous nucleation will improve the ability to design heteronuclear seeds and experiments to generate computationally predicted polymorphs.^53^

Hence, although the crystal energy landscape currently tends to over-predict polymorphism, modern solid form screening methods probably underestimate the range of polymorphs.^8^ There are inadequacies in the methods of calculating which structures are thermodynamically plausible as polymorphs, and a lack of understanding of how the kinetic factors of nucleation and growth can be varied by heterogeneous nucleation and the extent to which we can vary the conditions to find new forms. At least, if a structure has been shown by reliable CSP to be thermodynamically plausible, the question is what experiment might nucleate that form for the first time or at least help determine that it is experimentally unreachable, given the target structure. This is a significant advance on empirical polymorph screening.

## Conclusions

Recent advances in our ability to calculate worthwhile crystal energy landscapes for larger molecules have enabled them to be combined with industrial quality experimental solid form screening results. This has shown that the crystal energy landscape gives a useful framework for understanding the complexity of solid form landscapes for small drug molecules, and has the potential to help direct effective experimentation. There is no routine “black-box” recipe for either computational (CSP) or experimental polymorph screening, with both needing adapting to the properties of the individual molecule and the aim of the study. However, the successes of computed crystal energy landscapes for motivating the finding of thermodynamically stable polymorphs, helping to structurally characterise new polymorphs, anticipating disorder and generally helping to rationalise the diversity of the solid form landscapes, shows that such methods can form a valuable complement to solid form screening.
